# ATP regulates RNA‐driven cold inducible RNA binding protein phase separation

**DOI:** 10.1002/pro.4123

**Published:** 2021-05-22

**Authors:** Qishun Zhou, Sinem Usluer, Fangrong Zhang, Aneta J. Lenard, Benjamin M. R. Bourgeois, Tobias Madl

**Affiliations:** ^1^ Gottfried Schatz Research Center for Cell Signaling, Metabolism and Aging, Molecular Biology & Biochemistry Medical University of Graz Graz Austria; ^2^ BioTechMed‐Graz Graz Austria

**Keywords:** ATP, CIRBP, disordered protein, liquid–liquid phase separation, RG/RGG region, RNA‐binding protein

## Abstract

Intrinsically disordered proteins and proteins containing intrinsically disordered regions are highly abundant in the proteome of eukaryotes and are extensively involved in essential biological functions. More recently, their role in the organization of biomolecular condensates has become evident and along with their misregulation in several neurologic disorders. Currently, most studies involving these proteins are carried out in vitro and using purified proteins. Given that in cells, condensate‐forming proteins are exposed to high, millimolar concentrations of cellular metabolites, we aimed to reveal the interactions of cellular metabolites and a representative condensate‐forming protein. Here, using the arginine–glycine/arginine–glycine–glycine (RG/RGG)‐rich cold inducible RNA binding protein (CIRBP) as paradigm, we studied binding of the cellular metabolome to CIRBP. We found that most of the highly abundant cellular metabolites, except nucleotides, do not directly bind to CIRBP. ATP, ADP, and AMP as well as NAD^+^, NADH, NADP^+^, and NADPH directly interact with CIRBP, involving both the folded RNA‐recognition motif and the disordered RG/RGG region. ATP binding inhibited RNA‐driven phase separation of CIRBP. Thus, it might be beneficial to include cellular metabolites in in vitro liquid–liquid phase separation studies of RG/RGG and other condensate‐forming proteins in order to better mimic the cellular environment in the future.

**Abbreviations**
CIRBP: cold inducible RNA binding protein
CSP: chemical shift perturbation
DIC: differential interference contrast
FUS: fused in sarcoma
HSQC: heteronuclear single quantum coherence
IDP: intrinsically disordered protein
IDR: intrinsically disordered region
LLPS: liquid–liquid phase separation
NMR: nuclear magnetic resonance
NOE: nuclear Overhauser enhancement
RBPs: RNA‐binding protein
RG/RGG: arginine–glycine/arginine–glycine–glycine
RNP: ribonucleoprotein
RRM: RNA‐recognition‐motif
RSY: arginine–serine–tyrosine
SG: stress granule

## 1 INTRODUCTION

Intrinsically disordered proteins (IDPs) and proteins harboring intrinsically disordered regions (IDRs) constitute 33% of the eukaryotic and 44% of the human proteome.^1^, ^2^, ^3^ Their functional implications include essential biological processes like cell signaling,^4^, ^5^ gene transcription,^6^ RNA splicing,^7^, ^8^ and cell cycle regulation.^9^, ^10^ Over the last decades, a number of studies have demonstrated the essential role of IDPs/IDRs in the formation of biomolecular condensates also referred as membraneless organelles.^4^, ^11^, ^12^ Ribonucleoprotein (RNP) granules, such as stress granules (SGs), P‐bodies, Cajal bodies, or paraspeckles constitute a large class of these condensates and are composed of a dynamic assembly of RNA and RNA‐binding proteins (RBPs).^13^, ^14^, ^15^ IDRs in RBPs can undergo liquid–liquid phase separation (LLPS) in vitro which constitute a physical mechanism allowing the formation of these biological assemblies with dynamic, liquid‐like behavior in vivo.^14^, ^16^, ^17^ Mutations in these regions are often associated with aberrant localization of RBPs and the formation of pathological aggregates,^14^, ^18^, ^19^ and associate with a large variety of diseases including cancers and neurodegenerative diseases.^18^, ^20^ Therefore, these RBPs are considered as potential therapeutic targets and, thus, there is a need for a better understanding of the cellular mechanisms involved in the regulation of the formation of RNP granules in vivo as well as RBPs LLPS in vitro. Cellular metabolites are one of the most abundant molecules in living cells with a total intracellular concentration of about 200 mM in mammalian iBMK cells.^21^ Therefore, in this study we addressed the question whether some of these highly abundant metabolites could regulate LLPS of RBPs in vitro.

Arginine–glycine/arginine–glycine–glycine (RG/RGG) rich regions constitute a large fraction of IDRs in RBPs and have been shown to contribute to LLPS in vitro and associate with several biomolecular condensates in vivo,^22^, ^23^ respectively. Furthermore, recent studies demonstrated that adenine triphosphate (ATP), a highly abundant cellular metabolite, can directly bind to the RG/RGG region, as well as the RNA‐recognition‐motif (RRM) of the RBP fused in sarcoma (FUS), thereby affecting both FUS LLPS and fibrillirization.^24^, ^25^ Whether ATP regulates phase separation of other RBPs containing RG/RGG regions and whether this is a general mechanism controlling their association with RNP granules is still unclear. Furthermore, whether other metabolites bind RBPs and share similar LLPS‐modulatory functions as ATP remains unknown.

**FIGURE 1:**
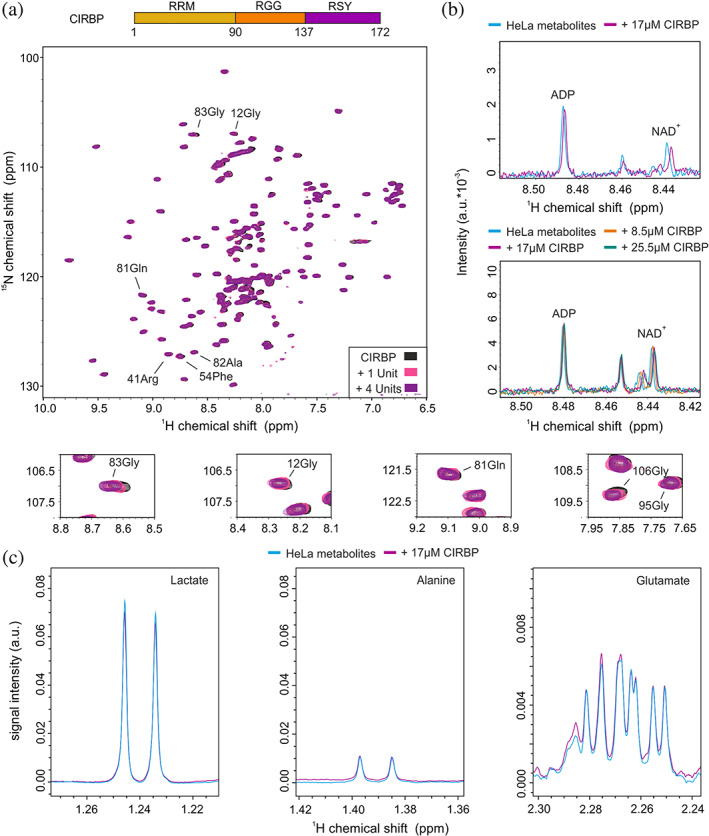
HeLa Cell metabolites contain binding partners of CIRBP. (a) ^1^H‐^15^N HSQC spectrum of ^15^N‐labeled CIRBP at 17 μM without (black) or with addition of 1 or 4 units of HeLa metabolites extract (pink and magenta, respectively). Overlay of 1D ^1^H‐CPMG spectra of HeLa metabolites extract in absence (cyan) or presence of increasing concentrations of CIRBP ranging from 8.5 to 25.5 μM (orange, magenta and green) and zoomed in a region corresponding to ADP and NAD+ in (b) and lactate, alanine and glutamate in (c)

We recently showed that the RG/RGG region of the cold inducible RNA binding protein (CIRBP) is essential to mediate both CIRBP phase separation in vitro and stress‐granule association in cells.^26^ We therefore used CIRBP as model system to study the influence of human cell metabolites on CIRBP LLPS. CIRBP consists of a conserved folded RNA recognition motif (RRM, 1–90), a disordered RG/RGG region (90–137) and a disordered arginine–serine–tyrosine (RSY) rich region (138–172) (Figure 1a). CIRBP belongs to the family of cold shock proteins.^27^, ^28^ In response to cellular stresses like ultraviolet irradiation, mild cold shock and hypoxia, the expression level of CIRBP is upregulated^29^, ^30^, ^31^ and CIRBP is translocated from the nucleus to the cytoplasm where it associates with SGs.^32^ Cytoplasmic CIRBP stabilizes and protects mRNA from stress conditions.^33^, ^34^ In addition, it is involved in cancer development by acting both as tumor promotor and tumor suppressor in different biological contexts.^35^

Using nuclear magnetic resonance (NMR) spectroscopy we showed that among the most abundant cellular metabolites, only ATP, ADP, and AMP (nucleotides) and NAD+, NADH, NADP+, and NADPH (dinucleotides) directly bind to CIRBP and that this involved both the RRM as well as the RG/RGG rich region. Using turbidity assays and differential interference contrast (DIC) microscopy we showed that ATP binding prevents CIRBP LLPS by competing with RNA‐driven RG/RGG phase separation. Summarizing, our study provides new mechanisms involved in the regulation of phase separation of RBPs as well as a better comprehension of the role of cellular metabolites involved in such processes.

## 2 RESULTS

### 2.1 HeLa cell metabolites contain binding partners of CIRBP

In order to validate our hypothesis that CIRBP can interact with cellular metabolites, we tested binding of extracted HeLa cell metabolites to a solution of recombinant and isotope labeled CIRBP using NMR spectroscopy. Stepwise addition of HeLa metabolites extract resulted in progressive chemical shift perturbations (CSPs) of several CIRBP ^1^H‐^15^N HSQC cross‐peaks (Figure 1a), indicating the direct interaction between CIRBP and some yet undefined HeLa metabolites. To determine which metabolites are specifically involved in the interaction, we further performed NMR analysis of HeLa metabolites extract upon addition of CIRBP. The analysis revealed minor CSPs of ^1^H metabolite peaks upon stepwise addition of CIRBP. The affected peaks were assigned to the ^1^H peaks corresponding to ADP as well as NAD^+^ (Figure 1b). Most of the other metabolite ^1^H peaks remained unaffected upon addition of CIRBP, including those of the most abundant metabolites lactate, alanine and glutamate (Figure 1c). This shows that the majority of Hela metabolites do not contribute to CIRBP binding aside from a specific subset including the nucleotides ADP and NAD^+^.

### 2.2 CIRBP interacts directly with (di)nucleotides, but none of the other highly abundant cellular metabolites

**FIGURE 2:**
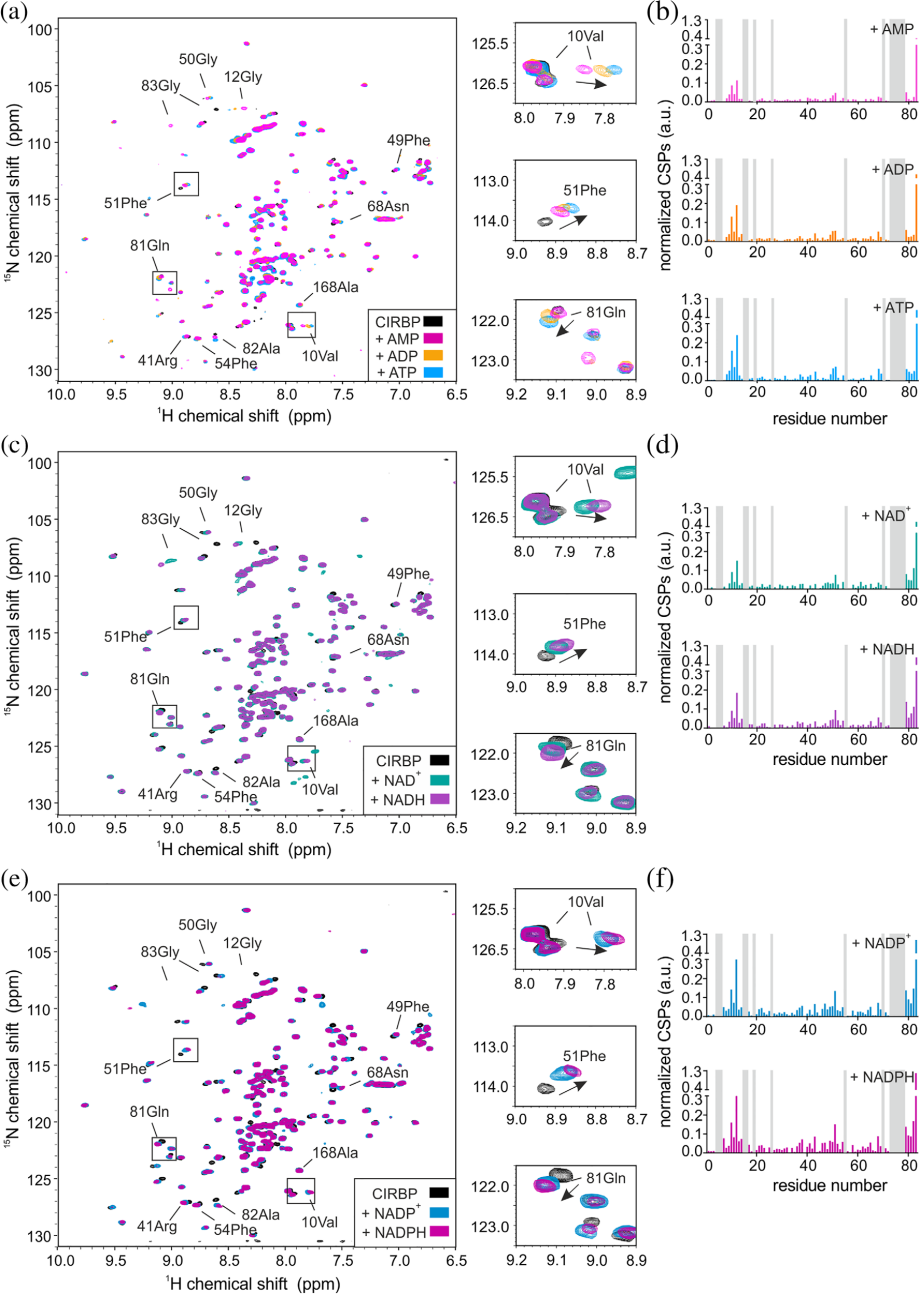
CIRBP directly interacts with (di)nucleotides. (a) ^1^H‐^15^N HSQC spectrum of ^15^N‐labeled CIRBP at 17 μM without (black) or with addition of 10 mM of either AMP, ADP, or ATP (pink, orange, and cyan, respectively). Chemical shift perturbations (CSPs) of the ^1^H‐^15^N HSQC CIRBP cross‐peaks between the CIRBP free and either AMP, ADP or ATP bound forms are shown in a bar‐plot in (b). Unassigned residues are indicated in grey. The CIRBP region between amino‐acids 91–172 is not shown as most of the corresponding ^1^H‐^15^N HSQC CIRBP cross‐peaks are unassigned. (c) ^1^H‐^15^N HSQC spectrum of ^15^N‐labeled CIRBP at 17 μM without (black) or with addition of 10 mM of either NAD^+^ or NADH (green and violet, respectively). CSPs of ^1^H‐^15^N HSQC CIRBP cross‐peaks between free CIRBP and either NAD^+^‐ or NADH‐bound forms are shown in bar‐plots in (d). Unassigned residues are indicated in grey. The CIRBP region between amino‐acids 91–172 is not shown as most of the corresponding ^1^H‐^15^N HSQC CIRBP cross‐peaks are unassigned. (e) ^1^H‐^15^N HSQC spectrum of ^15^N‐labeled CIRBP at 17 μM without (black) or upon addition of 10 mM of either NADP^+^ or NADPH (blue and magenta, respectively). CSPs of the ^1^H‐^15^N HSQC CIRBP cross‐peaks between free and either NADP^+^ or NADPH bound forms are shown in a bar‐plot in (f). Unassigned residues are indicated in grey. The CIRBP region between amino‐acids 91–172 is not shown as most of the corresponding ^1^H‐^15^N HSQC CIRBP cross‐peaks are unassigned. CIRBP, cold inducible RNA binding protein

**FIGURE 3:**
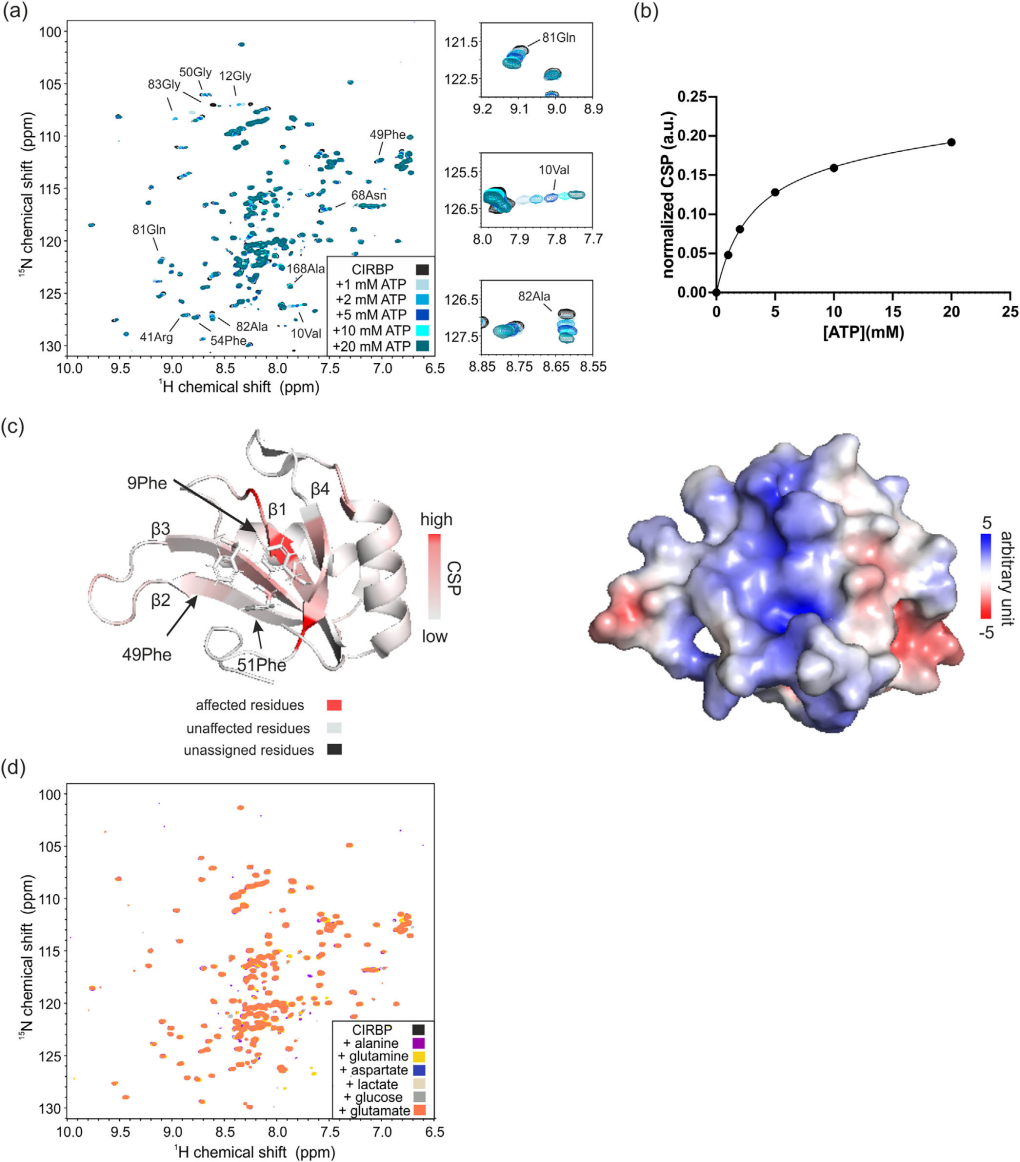
ATP binding to CIRBP overlaps with the putative RNA‐binding interface. (a) ^1^H, ^15^N HSQC spectrum of ^15^N‐labeled CIRBP at 17 μM without (black) or with increasing ATP concentration, ranging from 1 to 20 mM (blue gradient). (b) CSP plot of CIRBP ^1^H‐^15^N cross‐peak corresponding to valine 10 as function of ATP concentration. Using GraphPad Prism 8.4 we estimated the corresponding affinity with an associated K_D_ of 2.9 ± 1.4 mM using a nonlinear one binding site fitting function. (c) Mapping of the CSPs of CIRBP^1^H‐^15^N cross‐peaks (residue 1–90) upon binding to 10 mM ATP on the published structure of the RRM of CIRBP (PDB code: 5TBX). The electrostatic potential surface representation of the CIRBP^RRM^ is shown. (d) ^1^H‐^15^N HSQC spectrum of ^15^N‐labeled CIRBP at 17 μM without (black) or with addition of 3.3 mM of aspartate or 10 mM of either alanine, glutamine, lactate, glucose or glutamate (blue, magenta, yellow, light grey, grey, and orange, respectively). CIRBP, cold inducible RNA binding protein

In order to validate our results, we screened the most abundant metabolites in mammalian cells for CIRBP binding. It has been previously shown that among them and across organisms, amino‐acids followed by central carbon metabolites and nucleotides are by far the most abundant with concentrations up to 64 mM for glutamate.^21^ Although lactate concentrations were not included in these studies, it is among the most abundant metabolites in cells and included in our study. Consistent with our results obtained for the HeLa metabolite extract, ADP and NAD^+^ showed binding to CIRBP (Figure 2a,c). Moreover, the other tested nucleotides (ATP, AMP) as well as dinucleotides (NADH, NADP^+^ and NADPH) showed significant binding to CIRBP, as demonstrated by CSPs of several CIRBP ^1^H‐^15^N cross‐peaks upon addition of the respective nucleotides (Figure 2a,c,e). It is worth noting, that the same set of signals is shifted upon (di)nucleotide addition, with ATP and NADPH showing the strongest shifts. This indicates that the binding mode is similar between these (di)nucleotides and that ATP and NADPH have the highest affinity for CIRBP (Figure 2b,d,f). ATP binding to CIRBP is in the fast exchange regime with an associated K_D_ of 2.9 ± 1.4 mM (Figure 3a,b). The highest CSPs are associated with the CIRBP residues 10Val, 12Gly, 51Phe, 81Gln, and 83Gly and most of the affected and assigned CIRBP ^1^H‐^15^N cross‐peaks correspond to amino‐acids clustering within a positively charged surface comprising β‐strands β1, β3, and β4 of the CIRBP RRM (Figure 3c). Interestingly, 83Gly is the most affected CIRBP amino acid both with isolated (di)nucleotides and HeLa metabolite extract (Figures 1a and 2a,c,e), confirming that the CSPs observed upon addition of HeLa metabolites extract are mainly due to the (di)nucleotide components. In agreement, addition of the other highly abundant cellular metabolites, including alanine, aspartate, glutamine, glutamate, lactate, and glucose did not change the ^1^H‐^15^N HSQC spectrum of CIRBP, indicating that none of them interact with CIRBP with a comparable or higher affinity (Figure 3d).

Summarizing we showed that among the most concentrated cellular metabolites, only nucleotides binds directly to CIRBP whereas amino acids and central carbon metabolites do not show any binding.

### 2.3 The RRM and RG/RGG region of CIRBP are involved in (di)nucleotide binding

**FIGURE 4:**
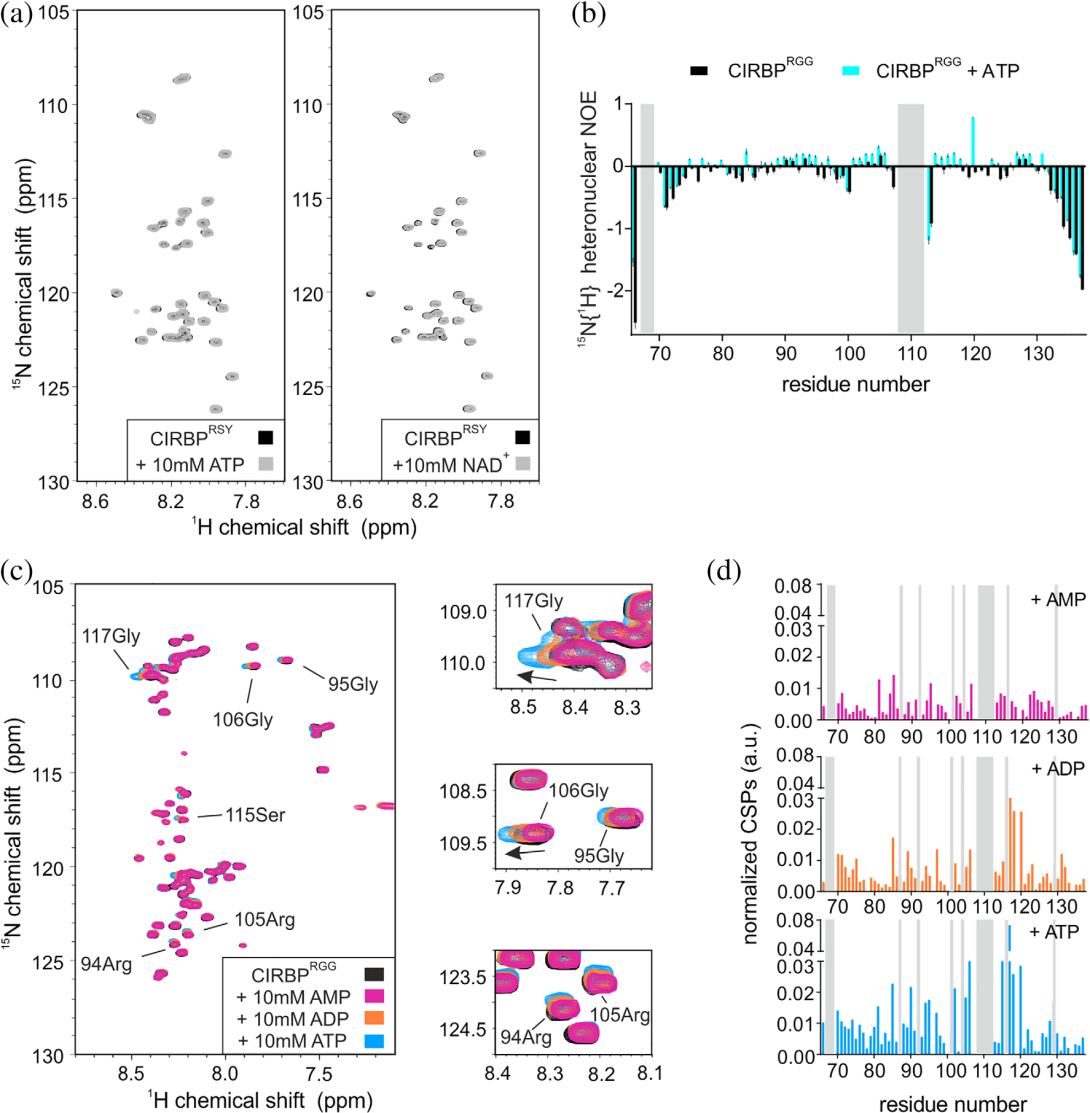
The RG/RGG region of CIRBP are involved in nucleotide binding. (a) ^1^H‐^15^N HSQC spectrum of ^15^N‐labeled CIRBP^RSY^ at 30 μM without (black) or with addition of 10 mM of ATP or NAD^+^ (grey, left and right panel, respectively). (b) ^1^H‐^15^N HSQC spectrum of ^15^N‐labeled CIRBP^RGG^ at 30 μM without (black) or with addition of 10 mM of either AMP, ADP, or ATP (magenta, orange, and blue, respectively). CSPs of the ^1^H‐^15^N HSQC CIRBP^RGG^ cross‐peaks between the CIRBP free and either AMP, ADP or ATP bound forms are shown in a bar‐plot in (c). Unassigned residues are indicated in grey. (d) ^15^N{^1^H} heteronuclear NOE values are plotted versus CIRBP^RGG^ residue numbers in presence (cyan) or absence (black) of ATP. Error bars for heteronuclear NOE values were derived from error propagation calculation using standard deviation of 10 arbitrarily chosen noise peaks in saturated and unsaturated spectra. CIRBP, cold inducible RNA binding protein

**FIGURE 5:**
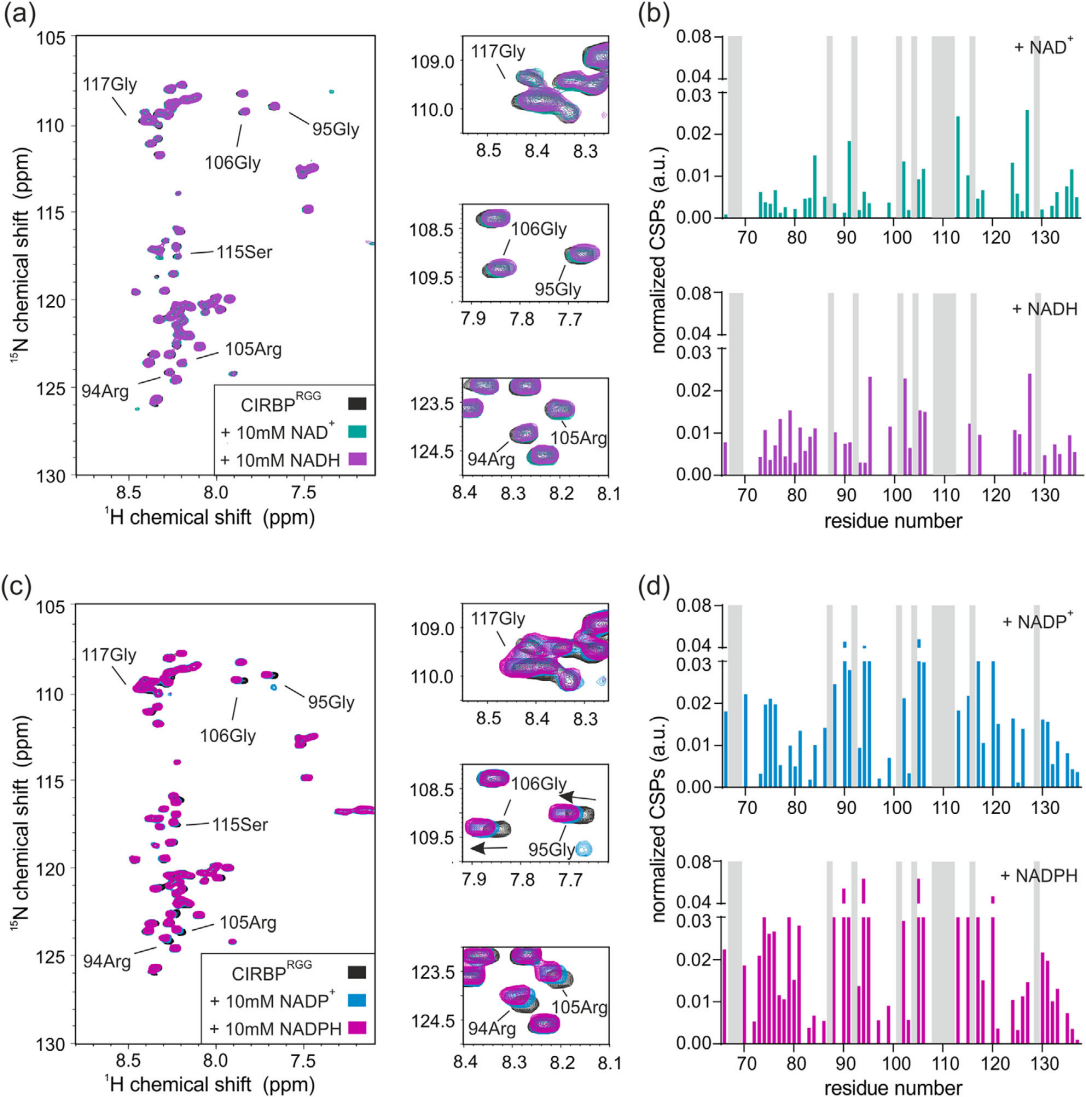
The RG/RGG region of CIRBP is involved in dinucleotide binding. (a) ^1^H‐^15^N HSQC spectrum of ^15^N‐labeled CIRBP^RGG^ at 30 μM without (black) or upon addition of 10 mM of either NAD^+^ or NADH (green and violet, respectively). CSPs of ^1^H‐^15^N HSQC CIRBP^RGG^ cross‐peaks between free and either NAD^+^ or NADH bound forms are shown in a bar‐plot in (b). The unassigned residues are indicated in grey. (c) ^1^H‐^15^N HSQC spectrum of ^15^N‐labeled CIRBP^RGG^ at 30 μM without (black) or upon addition of 10 mM of either NADP^+^ or NADPH (blue and magenta, respectively). CSPs of ^1^H‐^15^N HSQC CIRBP^RGG^ cross‐peaks between free and either NADP^+^ or NADPH bound forms are shown in a bar‐plot in (d). unassigned residues are indicated in grey

**FIGURE 6:**
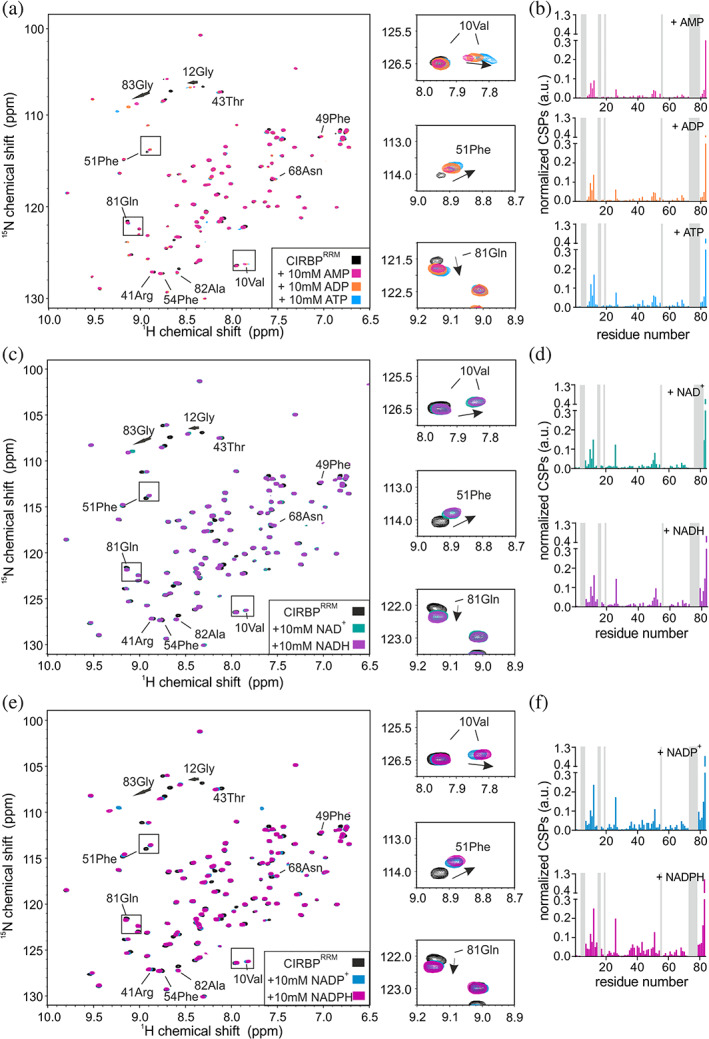
The RRM of CIRBP is involved in (di)nucleotide binding. (a) ^1^H‐^15^N HSQC spectrum of ^15^N‐labeled CIRBP^RRM^ at 30 μM without (black) or upon addition of 10 mM of either AMP, ADP or ATP (magenta, orange, and blue, respectively). CSPs of ^1^H‐^15^N HSQC CIRBP^RRM^ cross‐peaks between free and either AMP, ADP or ATP bound forms are shown in a bar‐plot in (b). Unassigned residues are indicated in grey. (c) ^1^H‐^15^N HSQC spectrum of ^15^N‐labeled CIRBP^RRM^ at 30 μM without (black) or upon addition of 10 mM of either NAD^+^ or NADH (green and violet, respectively). CSPs of ^1^H‐^15^N HSQC CIRBP^RRM^ cross‐peaks between free and either NAD^+^ or NADH bound forms are shown in a bar‐plot in (d). Unassigned residues are indicated in grey. (e) ^1^H‐^15^N HSQC spectrum of ^15^N‐labeled CIRBP^RRM^ at 30 μM without (black) or upon addition of 10 mM of either NADP^+^ or NADPH (blue and magenta, respectively). CSPs of ^1^H‐^15^N HSQC CIRBP^RRM^ cross‐peaks between free and either NADP^+^ or NADPH bound forms are shown in a bar‐plot in (f). Unassigned residues are indicated in grey. CIRBP, cold inducible RNA binding protein

To explore how independent regions of CIRBP are involved in (di)nucleotide binding, we purified isolated CIRBP regions, CIRBP^RRM^ (1–90), CIRBP^RGG^ (90–137) and CIRBP^RSY^ (138–172) and tested their binding to (di)nucleotides using NMR. Addition of ATP and NAD^+^ to a solution of isotope labeled CIRBP^RSY^ did not affect the ^1^H‐^15^N HSQC spectrum, indicating that the CIRBP RSY region is not involved in ATP and NAD^+^ binding (Figure 4a). In contrast, upon addition of (di)nucleotides, we observed CSPs of several CIRBP^RGG^ as well as CIRBP^RRM 1^H‐^15^N cross‐peaks (Figures 4b,c, 5a–d, and 6a–f), indicating direct interaction between both the RRM and the RG/RGG region of CIRBP with these (di)nucleotides. In case of AMP, NAD^+^ and NADH, CSPs of CIRBP^RGG 1^H‐^15^N cross‐peaks are weak and distributed along the entire RG/RGG region. This suggests unspecific binding of AMP, NAD^+^, and NADH to the CIRBP RG/RGG region. As for CIRBP full‐length, the same set of signals of the RRM were shifted upon (di)nucleotide addition, with ATP and NADPH showing the strongest effect (Figure 6b,d,e). This indicates that the binding mode is similar between these nucleotides for both CIRBP full length and the isolated RRM, with ATP and NADPH harboring the strongest affinity. Furthermore, using ^15^N{^1^H} heteronuclear NOE experiments of isotope labeled CIRBP^RGG^ in presence or absence of ATP, we observed higher heteronuclear NOE values of a set of CIRBP^RGG 1^H‐^15^N cross‐peaks upon ATP addition indicating increased rigidity of the RG/RGG region in the ATP bound state (Figure 4d).

Summarizing, we showed that both the CIRBP RRM as well as the RG/RGG region can independently interact with (di)nucleotides.

### 2.4 ATP binding to the CIRBP RG/RGG region attenuates RNA binding and is regulated by arginine methylation

**FIGURE 7:**
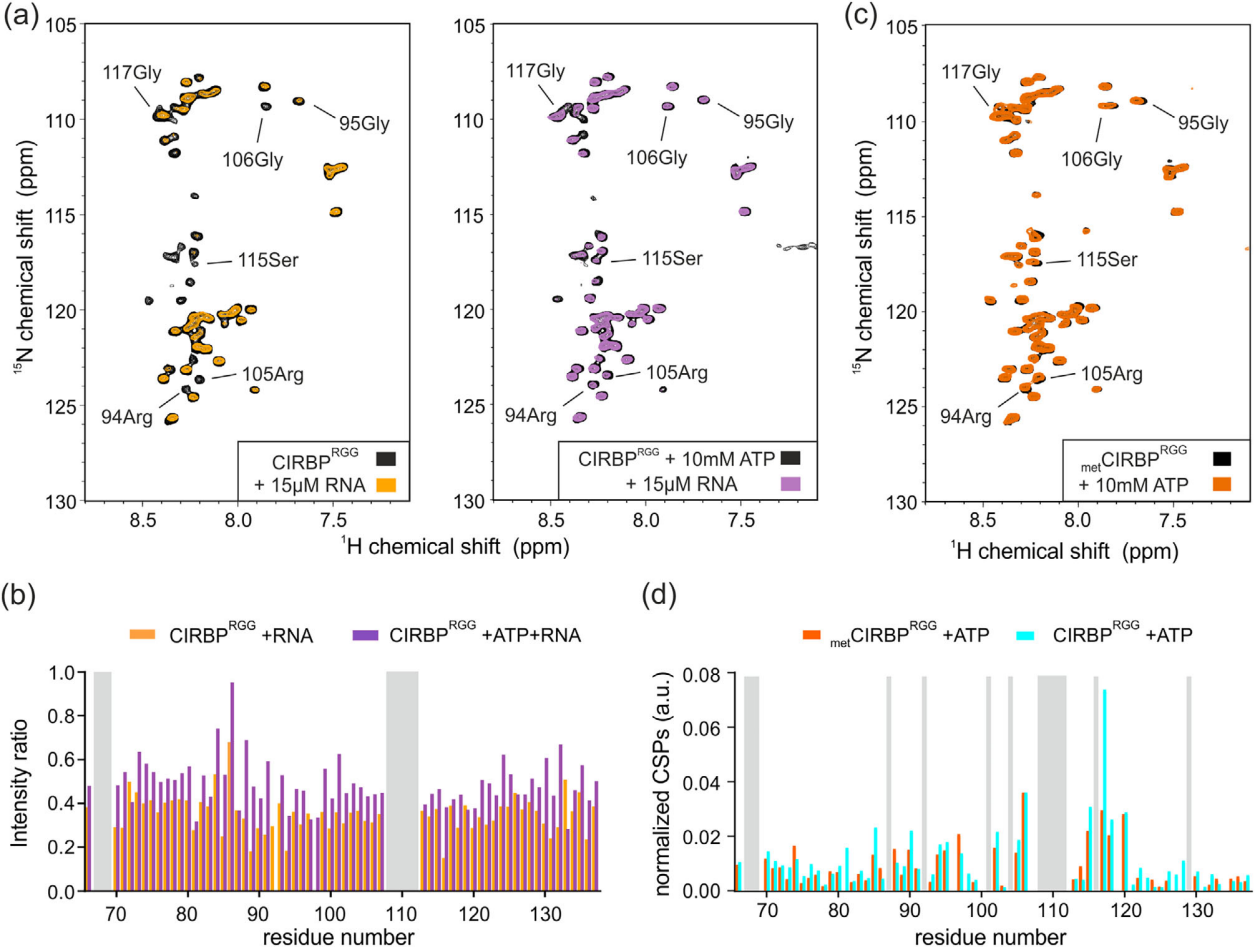
ATP binding to the CIRBP RG/RGG region attenuates RNA binding and is regulated by arginine methylation. (a) ^1^H‐^15^N HSQC spectrum of ^15^N‐labeled CIRBP^RGG^ at 30 μM in presence or absence of 10 mM ATP (right and left panel, respectively) and without (black) or with addition of 15 μM (UG)_12_ RNA (orange, violet). The intensity ratio of the ^1^H‐^15^N HSQC CIRBP^RGG^ cross‐peaks between the ATP (orange) or ATP/RNA (violet) free and bound forms are shown in a bar‐plot in (b). The unassigned residues are indicated in grey. (c) ^1^H‐^15^N HSQC spectrum of ^15^N‐labeled _met_CIRBP^RGG^ at 30 μM in presence (orange) or absence of 10 mM ATP (black). CSPs of the ^1^H‐^15^N HSQC methylated _met_CIRBP^RGG^ (orange) or unmethylated CIRBP^RGG^ (blue) cross‐peaks between the ATP free and bound forms are shown in a bar‐plot in (d). The unassigned residues are indicated in grey. CIRBP, cold inducible RNA binding protein

We previously showed that the RG/RGG region of CIRBP can directly interact with RNA.^26^ Given that both ATP and RNA are negatively charged, we hypothesized that they could share similar binding modes and compete together for CIRBP RG/RGG binding. We therefore performed an NMR‐based competition assay to access whether ATP competes with RNA while binding to CIRBP^RGG^. Line broadening of CIRBP^RGG 1^H‐^15^N cross‐peaks was observed upon addition of unlabeled (UG)_12_ RNA to a solution of isotope labeled CIRBP^RGG^, (Figure 7a, left panel) which indicates direct interaction between the CIRBP RG/RGG region and (UG)_12_ RNA, as previously shown.^26^ In contrast, presence of an excess of ATP leads to reappearance of the CIRBP^RGG 1^H‐^15^N cross‐peaks, indicating that ATP can compete with (UG)_12_ RNA for binding to the RG/RGG region of CIRBP in the experimental conditions tested (Figure 7a, right panel, Figure 7b).

As we and others, previously demonstrated that PRMT1‐mediated arginine methylation of the RG/RGG region regulates its binding to RNA,^36^ we next aimed to assess whether it can also influence ATP binding. We therefore in vitro methylated CIRBP RG/RGG using recombinant PRMT1, purified the methylated protein (_met_CIRBP^RGG^) and tested its binding to ATP by NMR spectroscopy. Addition of ATP to a solution of isotope labeled _met_CIRBP^RGG^ showed similar effects on the ^1^H‐^15^N HSQC spectrum of _met_CIRBP^RGG^ as compared to the same experiment performed with unmethylated CIRBP^RGG^, suggesting that methylation of the RG/RGG region of CIRBP has minor impact on its interaction with ATP molecules (Figure 7c,d).

### 2.5 ATP modulates LLPS of CIRBP

We previously showed that CIRBP can phase separate in vitro in the presence of RNA.^26^ As we have shown that ATP can efficiently compete with RNA for CIRBP binding, we hypothesized that ATP can also regulate its propensity to phase separate.

**FIGURE 8:**
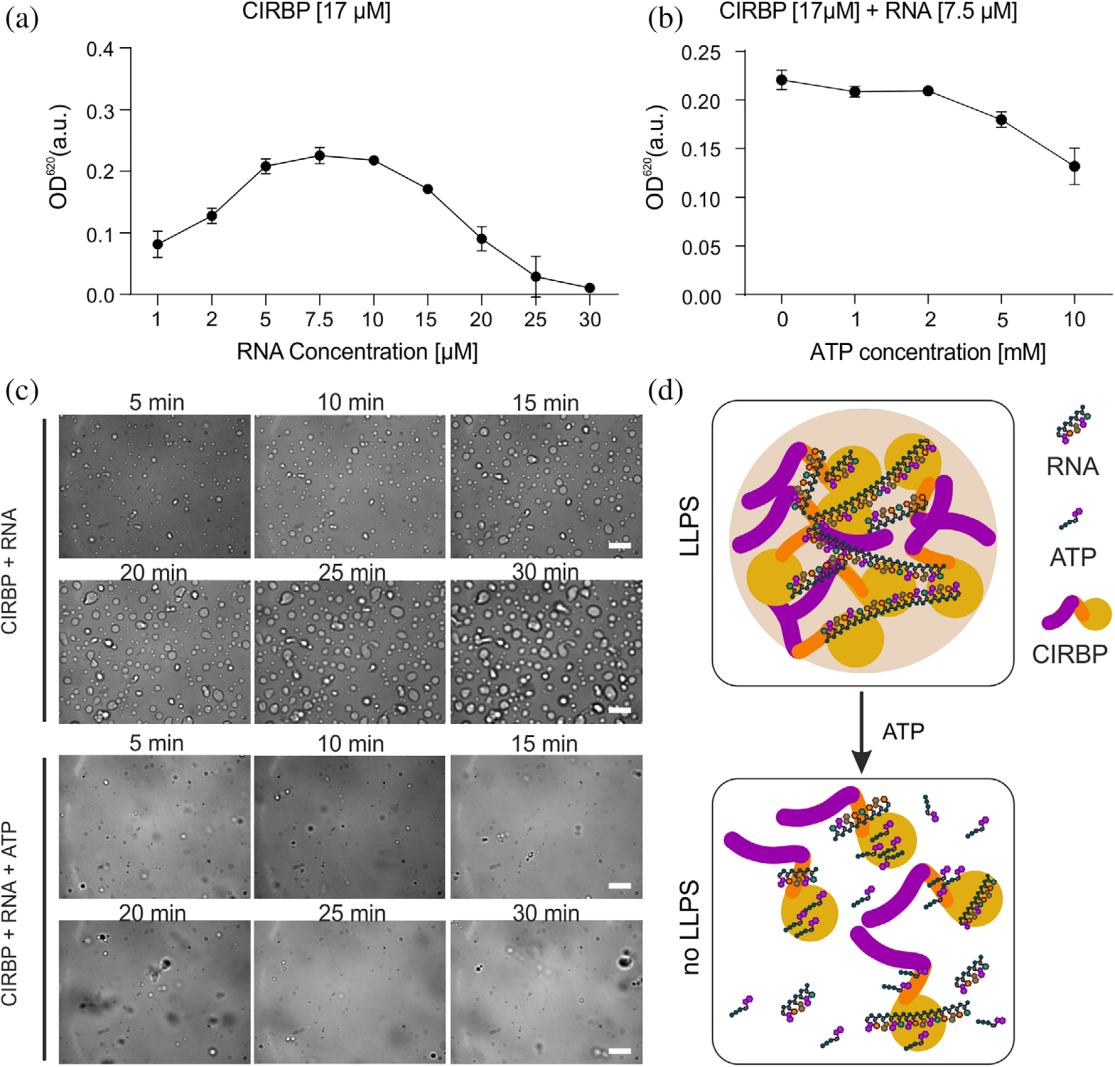
ATP modulates LLPS of CIRBP. Turbidity assay to quantify phase separation of full‐length CIRBP with fixed CIRBP concentration (17 μM) and increasing RNA concentration in (a), with fixed CIRBP and RNA concentration (17 and 7.5 μM, respectively) and increasing ATP concentration in (b). Values represent means ±SEM (*n* = 3). (c) DIC microscopy images of CIRBP at 17 μM in presence of 7.5 μM RNA and/or 5 mM ATP. Scale bar, 10 μm. (d) Model of ATP/RNA‐mediated regulation of CIRBP LLPS. CIRBP, cold inducible RNA binding protein; DIC, differential interference contrast; LLPS, liquid–liquid phase separation

To this end, we monitored phase separation of purified CIRBP in a turbidity assay, which uses the optical density of a protein solution as a measure of phase separation. In line with a previous study,^26^ titration of (UG)_12_ RNA to a sample with a fixed CIRBP concentration (17 μM) led to a progressive turbidity increase at low RNA concentrations, whereas higher amounts of RNA had a suppressive effect on CIRBP LLPS (Figure 8a). Addition of increasing amounts of ATP to RNA‐bound and phase separated CIRBP led to a concentration‐dependent decrease in turbidity, demonstrating that ATP inhibits CIRBP LLPS (Figure 8b). In order to further validate these findings, we monitored CIRBP phase separation by DIC microscopy. In the presence of substoichiometric amounts of RNA, CIRBP immediately formed small condensates that increased in size over time, most likely due to fusion of condensates, and indicating their “liquid‐like” behavior (Figure 8c). In line with the turbidity assay, addition of ATP was able to suppress CIRBP condensate formation (Figure 8c).

Summarizing, our data clearly demonstrate that ATP interferes with LLPS of CIRBP in vitro and likely with SGs recruitment in cells. This is probably due to interference between ATP and RNA‐mediated enhancement of CIRBP LLPS.

## 3 DISCUSSION

Here we showed using NMR spectroscopy that CIRBP, one of the constituent of SGs^26^, ^32^ directly interact with several (di)nucleotides present in HeLa cell metabolite extracts (Figure 1b). In contrast, other highly abundant metabolites which do not contain an aromatic moiety did not show any detectable binding to CIRBP despite their negatively charged nature. Hence, the carboxyl groups from lactate and other negatively charged amino acids are insufficient to mediate interaction with CIRBP, and possibly other RG/RGG‐containing proteins (Figure 1c). In addition, none of the other highly abundant metabolites alanine, glutamine, or glucose was found to bind to CIRBP. Therefore, our data suggest that both the aromatic and the phosphate moieties of (di)nucleotides contribute to efficient CIRBP interaction allowing recognition of this class of metabolites. To our knowledge, this is the first study demonstrating that the large majority of cellular metabolites do not bind RBP.

Here, we showed that both the RRM and the RG/RGG region of CIRBP can independently interact with (di)nucleotides (Figures 4b,c, 5, and 6). Binding of (di)nucleotides to the C‐terminal disordered RG/RGG region and RRM of CIRBP might involve (a) cation‐*π* interactions between adenosine aromatic cycles and arginines of the RG/RGG rich region or arginines/lysines of the RNA‐binding interface of the RRM,^23^, ^25^, ^37^ (b) electrostatic contacts between the phosphate groups and/or ribose sugars of the nucleotides and arginines/lysines,^23^, ^38^ (c) *π* − *π* stacking interactions between adenosine aromatic cycles and aromatic amino‐acids highly populated in the IDR regions of RBPs^37^, ^39^, ^40^ and a combination thereof. These interactions make (di)nucleotides probably divalent or multivalent ligands.^41^ Similar interactions have been shown for the RBP FUS, where ATP can also efficiently interact with both the RRM and the C‐terminal disordered RG/RGG region.^24^, ^25^ Therefore, it is tempting to speculate that (di)nucleotide binding to such RNA‐binding domains^25^ can be generalized to the large class of RBPs. The affinity of CIRBP for the tested (di)nucleotides can be estimated to be in the millimolar range. Among them, and to our knowledge, only ATP has been reported to reach millimolar intra‐cellular concentrations.^21^ Nevertheless, further studies are required to define the exact (sub)cellular, and context‐specific concentrations of these metabolites. Therefore, we cannot infer whether the observed binding for other tested metabolites would be functionally relevant in vivo.

Besides, we observed an increase in rigidity of the CIRBP RG/RGG region upon ATP binding (Figure 4d) implying that RG/RGG regions undergo local ordering in presence of nucleotides. Disordered proteins and nucleotides might originate earlier than folded proteins during evolution,^42^ nucleotides might thereupon act as folding chaperones, templates or inducers to enable disordered proteins reaching a suitable conformation for further interactions,^43^ as well as increasing solubility of unstructured proteins, thus counteracting their propensity to form pathological aggregates.^44^, ^45^, ^46^

In this regard, and besides its function as co‐factor for enzymes,^47^ ATP has more recently been shown to play a function as biological hydrotrope.^44^ Here, we showed, using turbidity assays and DIC microscopy, that ATP can efficiently dissolve CIRBP liquid droplets, thus interfering with CIRBP LLPS (Figure 8b,c). Such “chaperone” function of ATP has been shown for others RBPs, including FUS, TAF‐15 and hnRNPA3.^44^ These proteins share common features such as RG/RGG regions and RRMs, but the underlying ATP‐driven “chaperone” mechanisms remain unclear. CIRBP RRM possesses the features of a canonical RRM with a typical β1–α1–β2–β3–α2–β4 topology and three conserved aromatic side chains (9Phe, 49Phe, and 51Phe) located on β‐strand 1 and 3 which are essential for RNA binding (Figure 3c).^48^ The CIRBP RRM surface has been proposed, based on homology modeling, to mediate CIRBP binding to RNA.^49^ This is in agreement with the electrostatic potential surface of the CIRBP RRM showing a stretch of positively charged residues between the β‐strands β3 and β4 (Figure 3c). The same surface is responsible for ATP binding (Figure 3c). Therefore, it is tempting to speculate that ATP binding to the RRM will impair RNA binding.

Our data indicates that ATP‐mediated regulation of LLPS involves the CIRBP RG/RGG, but how this is linked to the in vivo situation remains unclear. We and others previously showed that RNA regulates LLPS of RG/RGG regions through the formation of intermolecular contacts.^23^, ^26^ Therefore, our data provide new mechanistic evidences suggesting that the intra‐cellular ATP/RNA ratio can influence RNP granule biogenesis, shifting the equilibrium toward granule formation or dissociation (Figure 8d). Concentrations of RNA and ATP in cells change upon cellular stress^50^ or aging.^24^ During oxidative stress, ATP is depleted from the cell and this correlates with CIRBP recruitment into SGs, a specific class of RNP granules.^32^, ^51^ Therefore, our findings provide first clues allowing a better understanding of the regulation of biomolecular condensates. They suggest that cellular concentration of nucleotides and derivatives govern intra‐cellular LLPS propensity of multiple RBPs containing RG/RGG regions via interfering with RNA binding. This might be of great biological interest as the mechanisms allowing RNP granule formation and dissociation are mis‐regulated in a plethora of diseases.

## 4 MATERIALS AND METHODS

### 4.1 Plasmids

Expression constructs for the fragments of wild type human CIRBP (1–172), CIRBP^RRM^ (1–90), CIRBP^RGG^ (68–137) and CIRBP^RSY^ (138–172) were generated by synthesis of the corresponding optimized CIRBP DNA constructs (Genscript) and insertion of these into pETM11‐His_6_‐protein A vector containing a Tobacco Etch Virus (TEV) protease cleavage site after protein A. The expression construct for the fragment of rat PRMT1 (11–353) in pET28b‐His_6_ vector was previously described.^52^

### 4.2 Protein expression and purification

For expression of recombinant unlabeled or ^15^N‐^13^C labeled His_6_‐protein A tagged constructs, the different bacterial expression vectors were transformed into *Escherichia coli (E.coli)*BL21‐DE3 Star strain and 1 L expression cultures were grown in lysogeny broth (LB) medium (for unlabeled proteins) or in minimal medium supplemented with 2 g of ^13^C_6_H_12_O_6_ (Cambridge Isotope Laboratories) and 1 g of ^15^NH_4_Cl (Sigma) (^15^N‐^13^C labeled proteins) at 37°C until OD (600)nm of 0.8 and further induced with 0.5 mM isopropyl β‐D‐1‐thiogalactopyranoside (IPTG) followed by protein expression for 16 hr at 20°C (CIRBP^RRM^, CIRBP^RGG^, CIRBP^RSY^ and PRMT1) or 1.5 hr at 37°C (CIRBP). Cell pellets were harvested and sonicated either in denaturating lysis buffer (50 mM Tris–HCl pH 7.5, 150 mM NaCl, 20 mM Imidazole, 6 M urea) for disordered protein fragments (CIRBP^RGG^ and CIRBP^RSY^) or in non‐denaturating lysis buffer (50 mM Tris–HCl pH 7.5, 150 mM NaCl, 20 mM imidazole, 2 mM tris(2‐carboxyethyl)phosphine [TCEP]) for folded protein fragments (CIRBP, CIRBP^RRM^ and PRMT1). For CIRBP^RRM^ and full‐length CIRBP, cell lysates were incubated for 30 min at room temperature with RNase A (Life Technologies) before centrifugation. Supernatants of His_6_‐protein A CIRBP, CIRBP^RRM^, CIRBP^RGG^, CIRBP^RSY^ were applied on a nickel‐nitrilotriacetic (Ni‐NTA) agarose resin (Qiagen) compacted in gravity columns and eluted with 50 mM Tris–HCl pH 7.5, 1 M NaCl, 500 mM imidazole, 2 mM TCEP, 0.04% NaN_3_ containing buffer. Eluted His_6_‐protein A tagged CIRBP and individual regions were cleaved with TEV protease overnight at 4°C. Untagged CIRBP was diluted 10 times in 50 mM Tris, pH 7.5, 6 M Urea containing buffer, isolated using a HiTrap Heparin HP affinity column (Cytiva) and eluted with a buffer containing 50 mM Tris, pH 7.5, 1 M NaCl, 6 M Urea. CIRBP was buffer exchanged in a 10X‐salt buffer containing 50 mM NaH_2_PO_4_/Na_2_HPO_4_, pH 6.5, 1,500 mM NaCl and further diluted 10 times in the same buffer without NaCl prior usage. Untagged CIRBP^RSY^, CIRBP^RGG^ and CIRBP^RRM^ were isolated using a second affinity purification using Ni‐NTA beads. A final size exclusion chromatography purification step was performed in the buffer of interest on a gel filtration column (Superdex 75 GE Healthcare for CIRBP^RRM^ and CIRBP^RGG^ and Superdex peptide, GE Healthcare for CIRBP^RSY^).

The supernatant of His_6_‐PRMT1 after cell lysis was applied on a 5 ml HisTrap HP column (GE Healthcare) at 4°C and eluted with 50 mM Tris–HCl, pH 7.5, 1 M NaCl, 500 mM imidazole, 2 mM TCEP, 0.04% NaN_3_ containing buffer. A final size exclusion chromatography purification step was performed in the buffer of interest on a gel filtration column (Superdex 200 GE Healthcare).

Protein concentrations were estimated from their absorbance at 280 nm, assuming that the ε at 280 nm was equal to the theoretical ε value.

### 4.3 Cell culture

HeLa cells (ATCC, Guernsey, UK) were seeded on 1.5H high precision glass cover slips (Marienfeld‐Superior) and cultured in DMEM supplemented with 2 mM glutamine, 1% PS (100 U/ml penicillin, 100 μg/ml streptomycin) and 10% fetal bovine serum (FBS). Cells were maintained in a humidified incubator at 37°C with 5% CO_2_.

### 4.4 Cell metabolite extraction

HeLa cells pellets were suspended in 2:1, ice‐cold methanol:H_2_O mixture, and transferred to a tube containing Precellys beads (1.4 mm zirconium oxide beads, Bertin Technologies, Villeurbanne, France) for homogenization by Precellys 24 tissue homogenizer (Bertin Technologies, Montigny‐le‐Bretonneux, France). After centrifugation at 13,000 rpm for 45 min (4°C), the supernatant was transferred to a fresh tube, and the samples were lyophilized at <1 Torr, 850 rpm, 25°C for 10 hr in a vacuum‐drying chamber (Savant Speedvac SPD210 vacuum concentrator), with an attached cooling trap (Savant RVT450 refrigerated vapor trap) and vacuum pump (VLP120) (Thermo Scientific, Waltham, MA). The lyophilized metabolites were redissolved into a buffer containing 50 mM NaH_2_PO_4_/Na_2_HPO_4_ at pH 6.5. The concentration of 10 μl redissolved metabolites extract diluted in 500 μl NMR sample was defined as one arbitrary concentration unit, which was measured to contain 126 μM lactate in three replicates.

### 4.5 Turbidity assay

CIRBP and RNA (12 × UG repeats) samples were prepared in 50 mM NaH_2_PO_4_/Na_2_HPO_4,_ pH 6.5, 150 mM NaCl. Turbidity measurements were conducted at 620 nm in 96‐well plates with 90 μl samples using a BioTek Power Wave HT plate reader (BioTek). Each experiment was performed in three replicates.

### 4.6 Differential interference contrast microscopy

CIRBP and RNA (12 × UG repeats) samples were prepared in 50 mM NaH_2_PO_4_/Na_2_HPO_4,_ pH 6.5, 150 mM NaCl. Thirty microliter of sample was plated on a 30 mm No. 1 round glass coverslip and mounted on an Observer D1 microscope with 100×/1.45 oil immersion objective (Zeiss). Protein droplets were viewed using HAL 100 halogen lamp, and images were captured with an OrcaD2 camera (Hamamatsu) using VisiView 4.0.0.13 software (Visitron Systems GmbH). Droplet formation was induced by addition of RNA.

### 4.7 In vitro arginine methylation

CIRBP^RGG^ and His_6_‐PRMT1 were equilibrated in methylation buffer containing 50 mM Na_2_HPO_4_/NaH_2_PO_4_, pH 8.0, 150 mM NaCl, 2 mM DTT. Fifty micromolar CIRBP^RGG^ was incubated with 25 μM His_6_‐PRMT1 in presence of 2 mM S‐Adenosylmethionine (New England biolabs) for 16 hr at room temperature. Untagged methylated CIRBP^RGG^ (_met_CIRBP^RGG^) was then isolated from His_6_‐PRMT1 performing a second affinity purification using Ni‐NTA beads and further equilibrated in the buffer of interest. _met_CIRBP^RGG^ was analyzed using NMR ^1^H‐^13^C HSQC spectrum to confirm the CIRBP methylation status.

### 4.8 NMR spectroscopy

All protein samples were equilibrated in buffer containing 50 mM NaH_2_PO_4_/Na_2_HPO_4_, 150 mM NaCl at pH 6.5. All spectral acquisitions were performed at 298 K on a 600 MHz Bruker Avance Neo NMR spectrometer equipped with a TXI 600S3 probehead. Spectra were processed using Topspin 4.1.

The Carr–Purcell–Meiboom–Gill (CPMG) pulse sequence was used to acquire ^1^H 1D NMR spectra with pre‐saturation for water suppression (cpmgpr1d, 512 scans, 73,728 points in F1, 11,904.762 Hz spectral width, 1,024 transients, recycle delay 4 s) for all HeLa metabolites binding analysis.

2D ^1^H‐^15^N HSQC pulse program using water flip‐back and preservation of equivalent pathways (PEP) (hsqcetfpf3gpsi, 64 scans for CIRBP and 32 scans for CIRBP fragments, 128 points in F1, 1,024 points in F2, 1949.318 Hz spectral width in F1, 9,615.385 Hz spectral width in F2, 1,024 transients) was used to analyze all protein binding and titrations studies.

Phase sensitive ge‐2D ^1^H,^15^N HSQC (hsqcnoef3gpsi, 16 scans, 128 points in F1, 1,024 points in F2, 1,278.772 Hz spectral width in F1, 9,615.385 Hz spectral width in F2, 1,024 transients) was used to acquire heteronuclear ^15^N{^1^H} NOE of CIRBP^RGG^. The ^15^N{^1^H} heteronuclear NOE experiments were recorded with a saturation period/total interscan delay of 3.0 s.

Normalized CSPs of CIRBP ^1^H‐^15^N cross‐peaks upon binding to (di)nucleotides were calculated according to the following equation:

$$\mathrm{CSP}=\sqrt{{\left(\delta_{H}\right)}^{2}+\frac{{\left(\delta_{N}\right)}^{2}}{10}},$$

where *δ*_*H*_ is the ^1^H chemical shift difference between CIRBP ^1^H‐^15^N cross‐peaks in the bound and free states and *δ*_*N*_ is the ^15^N chemical shift difference between CIRBP ^1^H‐^15^N cross‐peaks in the bound and free states.

## CONFLICT OF INTEREST

The authors declare no competing interests.

## AUTHOR CONTRIBUTIONS

**Qishun Zhou:** Data curation; formal analysis; investigation; methodology; resources; visualization; writing‐original draft. **Sinem Usluer:** Data curation; investigation; resources; validation. **Fangrong Zhang:** Formal analysis; resources; writing‐review & editing. **Aneta Lenard:** Methodology; resources; writing‐review & editing. **Benjamin Bourgeois:** Conceptualization; writing‐review & editing. **Tobias Madl:** Funding acquisition; supervision; writing‐review & editing.

## References

[1] Deiana A , Forcelloni S , Porrello A , Giansanti A . Intrinsically disordered proteins and structured proteins with intrinsically disordered regions have different functional roles in the cell. PLoS One. 2019;14:e0217889. 31425549 10.1371/journal.pone.0217889 PMC6699704

[2] Ward JJ , Sodhi JS , Mcguffin LJ , Buxton BF , Jones DT . Prediction and functional analysis of native disorder in proteins from the three kingdoms of life. J Mol Biol. 2004;337:635–645. 15019783 10.1016/j.jmb.2004.02.002

[3] Van Der Lee R , Buljan M , Lang B , et al. Classification of intrinsically disordered regions and proteins. Chem Rev. 2014;114:6589–6631. 24773235 10.1021/cr400525m PMC4095912

[4] Wright PE , Dyson HJ . Intrinsically disordered proteins in cellular signalling and regulation. Nat Rev Mol Cell Biol. 2015;16:18–29. 25531225 10.1038/nrm3920 PMC4405151

[5] Bürgi J , Xue B , Uversky VN , Van Der Goot FG . Intrinsic disorder in transmembrane proteins: Roles in signaling and topology prediction. PLoS One. 2016;11:e0158594. 27391701 10.1371/journal.pone.0158594 PMC4938508

[6] Lakoucheva LM , Brown CJ , Lawson JD , Obradović Z , Dunker AK . Intrinsic disorder in cell‐signaling and cancer‐associated proteins. J Mol Biol. 2002;323:573–584. 12381310 10.1016/s0022-2836(02)00969-5

[7] Hamann F , Schmitt A , Favretto F , et al. Structural analysis of the intrinsically disordered splicing factor Spp2 and its binding to the DEAH‐box ATPase Prp2. Proc Natl Acad Sci U S A. 2020;117:2948–2956. 31974312 10.1073/pnas.1907960117 PMC7022188

[8] Korneta I , Bujnicki JM . Intrinsic disorder in the human spliceosomal proteome. PLoS Comput Biol. 2012;8:e1002641. 22912569 10.1371/journal.pcbi.1002641 PMC3415423

[9] Ou L , Waddell MB , Kriwacki RW . Mechanism of cell cycle entry mediated by the intrinsically disordered protein p27Kip1. ACS Chem Biol. 2012;7:678–682. 22276948 10.1021/cb200487h PMC3331940

[10] Fahmi M , Ito M . Evolutionary approach of intrinsically disordered CIP/KIP proteins. Sci Rep. 2019;9:1575. 30733475 10.1038/s41598-018-37917-5 PMC6367352

[11] Lee C‐YS , Putnam A , Lu T , He S , Ouyang JPT , Seydoux G . Recruitment of mRNAs to P granules by condensation with intrinsically‐disordered proteins. Elife. 2020;9:e52896. 31975687 10.7554/eLife.52896 PMC7007223

[12] Buchan JR . mRNP granules. Assembly, function, and connections with disease. RNA Biol. 2014;11:1019–1030. 25531407 10.4161/15476286.2014.972208 PMC4615263

[13] Protter DSW , Parker R . Principles and properties of stress granules. Trends Cell Biol. 2016;26:668–679. 27289443 10.1016/j.tcb.2016.05.004 PMC4993645

[14] Lin Y , David M , Parker R . Formation and maturation of phase‐separated liquid droplets by RNA‐binding proteins. Mol Cell. 2015;60:208–219. 26412307 10.1016/j.molcel.2015.08.018 PMC4609299

[15] Souquere S , Mollet S , Kress M , Dautry F , Pierron G , Weil D . Unravelling the ultrastructure of stress granules and associated P‐bodies in human cells. J Cell Sci. 2009;122:3619–3626. 19812307 10.1242/jcs.054437

[16] Lin Y , Currie SL , Rosen MK . Intrinsically disordered sequences enable modulation of protein phase separation through distributed tyrosine motifs. J Biol Chem. 2017;292:19110–19120. 28924037 10.1074/jbc.M117.800466 PMC5704491

[17] Uversky VN . Intrinsically disordered proteins in overcrowded milieu: Membrane‐less organelles, phase separation, and intrinsic disorder. Curr Opin Struct Biol. 2017;44:18–30. 27838525 10.1016/j.sbi.2016.10.015

[18] Martinelli A , Lopes F , John E , Carlini C , Ligabue‐Braun R . Modulation of disordered proteins with a focus on neurodegenerative diseases and other pathologies. Int J Mol Sci. 2019;20:1322. 10.3390/ijms20061322 PMC6471803 30875980

[19] Vance C , Scotter EL , Nishimura AL , et al. ALS mutant FUS disrupts nuclear localization and sequesters wild‐type FUS within cytoplasmic stress granules. Hum Mol Genet. 2013;22:2676–2688. 23474818 10.1093/hmg/ddt117 PMC3674807

[20] Liu X , Chen J . Modulation of p53 transactivation domain conformations by ligand binding and cancer‐associated mutations. Pac Symp Biocomput. 2020;25:195–206. 31797597 PMC6934143

[21] Park JO , Rubin SA , Xu Y‐F , et al. Metabolite concentrations, fluxes and free energies imply efficient enzyme usage. Nat Chem Biol. 2016;12:482–489. 27159581 10.1038/nchembio.2077 PMC4912430

[22] Thandapani P , O'Connor TR , Bailey TL , Richard S . Defining the RGG/RG motif. Mol Cell. 2013;50:613–623. 23746349 10.1016/j.molcel.2013.05.021

[23] Chong PA , Vernon RM , Forman‐Kay JD . RGG/RG motif regions in RNA binding and phase separation. J Mol Biol. 2018;430:4650–4665. 29913160 10.1016/j.jmb.2018.06.014

[24] Kang J , Lim L , Song J . ATP enhances at low concentrations but dissolves at high concentrations liquid‐liquid phase separation (LLPS) of ALS/FTD‐causing FUS. Biochem Biophys Res Commun. 2018;504:545–551. 30205960 10.1016/j.bbrc.2018.09.014

[25] Kang J , Lim L , Song J . ATP binds and inhibits the neurodegeneration‐associated fibrillization of the FUS RRM domain. Commun Biol. 2019;2:223. 31240261 10.1038/s42003-019-0463-x PMC6586847

[26] Bourgeois B , Hutten S , Gottschalk B , et al. Nonclassical nuclear localization signals mediate nuclear import of CIRBP. Proc Natl Acad Sci U S A. 2020;117:8503–8514. 32234784 10.1073/pnas.1918944117 PMC7165476

[27] Nishiyama H , Itoh K , Kaneko Y , Kishishita M , Yoshida O , Fujita J . A glycine‐rich RNA‐binding protein mediating cold‐inducible suppression of mammalian cell growth. J Cell Biol. 1997;137:899–908. 9151692 10.1083/jcb.137.4.899 PMC2139845

[28] Zhong P , Huang H . Recent progress in the research of cold‐inducible RNA‐binding protein. Future Sci OA. 2017;3:FSO246. 29134130 10.4155/fsoa-2017-0077 PMC5674272

[29] Sheikh MS , Carrier F , Papathanasiou MA , et al. Identification of several human homologs of hamster DNA damage‐inducible transcripts. J Biol Chem. 1997;272:26720–26726. 9334257 10.1074/jbc.272.42.26720

[30] Wellmann S , Bührer C , Moderegger E , et al. Oxygen‐regulated expression of the RNA‐binding proteins RBM3 and CIRP by a HIF‐1‐independent mechanism. J Cell Sci. 2004;117:1785–1794. 15075239 10.1242/jcs.01026

[31] Wang X , Che H , Zhang W , et al. Effects of mild chronic intermittent cold exposure on rat organs. Int J Biol Sci. 2015;11:1171–1180.31. 26327811 10.7150/ijbs.12161 PMC4551753

[32] De Leeuw F , Zhang T , Wauquier C , Huez G , Kruys V , Gueydan C . The cold‐inducible RNA‐binding protein migrates from the nucleus to cytoplasmic stress granules by a methylation‐dependent mechanism and acts as a translational repressor. Exp Cell Res. 2007;313:4130–4144. 17967451 10.1016/j.yexcr.2007.09.017

[33] Pan F , Zarate J , Choudhury A , Rupprecht R , Bradley TM . Osmotic stress of salmon stimulates upregulation of a cold inducible RNA binding protein (CIRP) similar to that of mammals and amphibians. Biochimie. 2004;86:451–461. 15308334 10.1016/j.biochi.2004.06.006

[34] Zhou M , Yang W‐L , Ji Y , Qiang X , Wang P . Cold‐inducible RNA‐binding protein mediates neuroinflammation in cerebral ischemia. Biochim Biophys Acta. 1840;2014:2253–2261. 10.1016/j.bbagen.2014.02.027 PMC4061249 24613680

[35] Lujan DA , Ochoa JL , Hartley RS . Cold‐inducible RNA binding protein in cancer and inflammation. Wiley Interdiscip Rev RNA. 2018;9:e1462. 10.1002/wrna.1462 PMC5886743 29322631

[36] Hofweber M , Hutten S , Bourgeois B , et al. Phase separation of FUS is suppressed by its nuclear import receptor and arginine methylation. Cell. 2018;173:706–719. 29677514 10.1016/j.cell.2018.03.004

[37] Myers JC , Shamoo Y . Human UP1 as a model for understanding purine recognition in the family of proteins containing the RNA recognition motif (RRM). J Mol Biol. 2004;342:743–756. 15342234 10.1016/j.jmb.2004.07.029

[38] Alayyoubi M , Leser GP , Kors CA , Lamb RA . Structure of the paramyxovirus parainfluenza virus 5 nucleoprotein–RNA complex. Proc Natl Acad Sci U S A. 2015;112:E1792–E1799. 25831513 10.1073/pnas.1503941112 PMC4394319

[39] Baker CM , Grant GH . Role of aromatic amino acids in protein‐nucleic acid recognition. Biopolymers. 2007;85:456–470. 17219397 10.1002/bip.20682

[40] Wilson KA , Holland DJ , Wetmore SD . Topology of RNA–protein nucleobase–amino acid π–π interactions and comparison to analogous DNA–protein π–π contacts. RNA. 2016;22:696–708. 26979279 10.1261/rna.054924.115 PMC4836644

[41] Ruff KM , Dar F , Pappu RV . Ligand effects on phase separation of multivalent macromolecules. Proc Natl Acad Sci U S A. 2021;118:e2017184118. 33653957 10.1073/pnas.2017184118 PMC7958451

[42] Kulkarni P , Uversky V . Intrinsically disordered proteins and the Janus challenge. Biomolecules. 2018;8:179. 10.3390/biom8040179 PMC6315817 30567293

[43] Cafferty BJ , Fialho DM , Khanam J , Krishnamurthy R , Hud NV . Spontaneous formation and base pairing of plausible prebiotic nucleotides in water. Nat Commun. 2016;7:11328. 27108699 10.1038/ncomms11328 PMC4848480

[44] Patel A , Malinovska L , Saha S , et al. ATP as a biological hydrotrope. Science. 2017;356:753–756. 28522535 10.1126/science.aaf6846

[45] Rice AM , Rosen MK . ATP controls the crowd. Science. 2017;356:701–702. 28522495 10.1126/science.aan4223

[46] Alberti S , Hyman AA . Are aberrant phase transitions a driver of cellular aging? Bioessays. 2016;38:959–968. 27554449 10.1002/bies.201600042 PMC5108435

[47] Nagana Gowda GA , Abell L , Lee CF , Tian R , Raftery D . Simultaneous analysis of major coenzymes of cellular redox reactions and energy using ex vivo 1H NMR spectroscopy. Anal Chem. 2016;88:4817–4824. 27043450 10.1021/acs.analchem.6b00442 PMC4857157

[48] Daubner GM , Cléry A , Allain FH‐T . RRM–RNA recognition: NMR or crystallography…and new findings. Curr Opin Struct Biol. 2013;23:100–108. 23253355 10.1016/j.sbi.2012.11.006

[49] Coburn K , Melville Z , Aligholizadeh E , et al. Crystal structure of the human heterogeneous ribonucleoprotein A18 RNA‐recognition motif. Acta Cryst F. 2017;73:209–214. 10.1107/S2053230X17003454 PMC5379170 28368279

[50] Guillén‐Boixet J , Kopach A , Holehouse AS , et al. RNA‐induced conformational switching and clustering of G3BP drive stress granule assembly by condensation. Cell. 2020;181:346–361. 32302572 10.1016/j.cell.2020.03.049 PMC7181197

[51] Zhang X , Wu XQ , Lu S , Guo YL , Ma X . Deficit of mitochondria‐derived ATP during oxidative stress impairs mouse MII oocyte spindles. Cell Res. 2006;16:841–850. 16983401 10.1038/sj.cr.7310095

[52] Zhang X , Cheng X . Structure of the predominant protein arginine methyltransferase PRMT1 and analysis of its binding to substrate peptides. Structure. 2003;11:509–520. 12737817 10.1016/s0969-2126(03)00071-6 PMC4030380

